# O‐GlcNAcylation of protein kinase A catalytic subunits enhances its activity: a mechanism linked to learning and memory deficits in Alzheimer's disease

**DOI:** 10.1111/acel.12449

**Published:** 2016-02-03

**Authors:** Shutao Xie, Nana Jin, Jianlan Gu, Jianhua Shi, Jianming Sun, Dandan Chu, Liang Zhang, Chun‐ling Dai, Jin‐hua Gu, Cheng‐Xin Gong, Khalid Iqbal, Fei Liu

**Affiliations:** ^1^ Jiangsu Key Laboratory of NeuroregenerationCo‐innovation Center of NeuroregenerationNantong UniversityNantongJiangsu226001China; ^2^ Department of NeurochemistryInge Grundke‐Iqbal Research FloorNew York State Institute for Basic Research in Developmental DisabilitiesStaten IslandNew York10314USA

**Keywords:** Alzheimer's disease, learning and memory, O‐GlcNAcylation, protein kinase A

## Abstract

Alzheimer's disease (AD) is characterized clinically by memory loss and cognitive decline. Protein kinase A (PKA)‐CREB signaling plays a critical role in learning and memory. It is known that glucose uptake and O‐GlcNAcylation are reduced in AD brain. In this study, we found that PKA catalytic subunits (PKAcs) were posttranslationally modified by O‐linked *N*‐acetylglucosamine (O‐GlcNAc). O‐GlcNAcylation regulated the subcellular location of PKAcα and PKAcβ and enhanced their kinase activity. Upregulation of O‐GlcNAcylation in metabolically active rat brain slices by *O*‐(2‐acetamido‐2‐deoxy‐d‐glucopyranosylidenamino) *N*‐phenylcarbamate (PUGNAc), an inhibitor of *N*‐acetylglucosaminidase, increased the phosphorylation of tau at the PKA site, Ser214, but not at the non‐PKA site, Thr205. In contrast, in rat and mouse brains, downregulation of O‐GlcNAcylation caused decreases in the phosphorylation of CREB at Ser133 and of tau at Ser214, but not at Thr205. Reduction in O‐GlcNAcylation through intracerebroventricular injection of 6‐diazo‐5‐oxo‐l‐norleucine (DON), the inhibitor of glutamine fructose‐6‐phosphate amidotransferase, suppressed PKA‐CREB signaling and impaired learning and memory in mice. These results indicate that in addition to cAMP and phosphorylation, O‐GlcNAcylation is a novel mechanism that regulates PKA‐CREB signaling. Downregulation of O‐GlcNAcylation suppresses PKA‐CREB signaling and consequently causes learning and memory deficits in AD.

## Introduction

Alzheimer's disease (AD) is a chronic progressive neurodegenerative disorder characterized pathologically by extracellular deposits of β‐amyloid as neuritic plaques (Glenner *et al*., 1984) and intracellular neurofibrillary tangles consisting of abnormally hyperphosphorylated aggregates of the microtubule‐associated protein tau (Grundke‐Iqbal *et al*., 1986a,b). The major clinical symptom of AD is memory loss and recognition decline.

Protein kinase A (PKA) is a cyclic AMP (cAMP)‐dependent kinase. It is involved in regulation of a vast number of cellular processes including metabolism, gene expression, proliferation, differentiation, sperm motility, and ion channel conductivity as well as learning and memory (Skalhegg & Tasken, 2000; Shabb, 2001; Kandel, 2012; Taylor *et al*., 2012). The holoenzyme of PKA consists of a regulatory subunit dimer, and each regulatory subunit (R) is bound to a catalytic subunit (C). Under low levels of cAMP, the holoenzyme remains intact and is catalytically inactive. When the intracellular concentration of cAMP rises, cAMP binds to the two binding sites on each regulatory subunit, which leads to the release of the catalytic subunits. Free catalytic subunits catalyze the phosphorylation of substrate proteins in the cytoplasm and in the nucleus (Taylor *et al*., 2012).

PKA phosphorylates numerous proteins and regulates their function. CREB is a well‐studied target of PKA. The activated PKA moves into the nucleus, where it activates CREB by phosphorylating it at Ser133 (Gonzalez & Montminy, 1989). CREB, a ubiquitous transcriptional factor, is a key molecule for learning and memory and a core component of the molecular switch that converts short‐term memory to long‐term memory (Barco *et al*., 2003). Disturbance of CREB function has been shown to cause memory deficits in many animal models. The downregulation of PKA‐CREB signaling is believed to be responsible for the deficit of learning and memory in AD brain and in some mouse models of AD (Yamamoto‐Sasaki *et al*., 1999; Puzzo *et al*., 2005; Matsuzaki *et al*., 2006; Liang *et al*., 2007).

The activity of PKA catalytic subunits (PKAcs) is regulated not only by the binding of cAMP onto regulatory subunit, but also by their phosphorylation at Thr197, which is autophosphorylated or phosphorylated by phosphoinositide‐dependent kinase‐1 (Moore *et al*., 2002). For maximal activity, each catalytic subunit must also be either autophosphorylated or phosphorylated at Thr197, which helps orient catalytic residues in the active site (Steichen *et al*., 2010, 2012). However, knowledge of PKA's other posttranslational modification remains limited.

Protein O‐GlcNAcylation is a unique posttranslational modification of a protein with O‐linked *N*‐acetylglucosamine (GlcNAc), which regulates multiple cellular functions. This process is catalyzed by O‐GlcNAc transferase (OGT), and the O‐GlcNAc group on proteins can be removed by the catalysis of β‐*N*‐acetylglucosaminidase (O‐GlcNAcase, OGA). To date, it has been found that all O‐GlcNAcylated proteins are phospho‐proteins. O‐GlcNAcylation regulates the phosphorylation of the same protein in a reciprocal manner. We have previously found decreased O‐GlcNAcylation in AD brain as a result of lower glucose uptake (Liu *et al*., 2004, 2009). However, whether the downregulation of O‐GlcNAc leads to cognitive decline in AD is not well known. Here, we show that both PKAcα and PKAcβ are modified by O‐GlcNAc. O‐GlcNAcylation positively regulates the function of PKA in the phosphorylation of CREB and tau. These findings led us to postulate that downregulation of O‐GlcNAcylation due to lower glucose uptake may suppress PKA‐CREB signaling and consequently contribute to memory loss and cognitive decline in AD.

## Results

### PKAcs are modified by O‐GlcNAc

**Figure 1:**
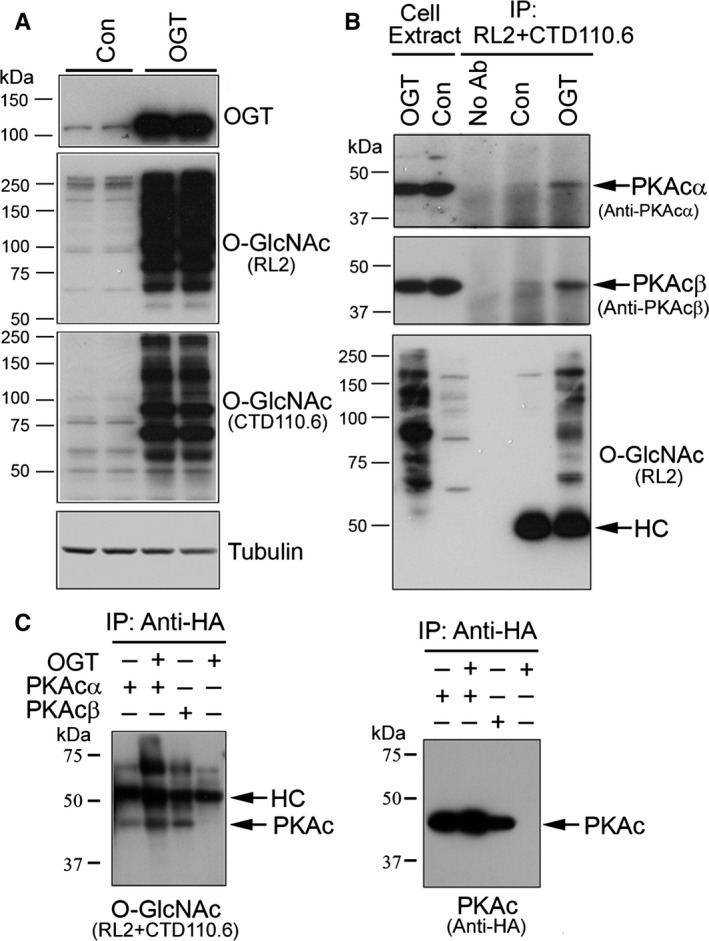
PKAcα and PKAcβ are modified by O‐GlcNAc. (A) Overexpression of OGT upregulated protein O‐GlcNAcylation. HEK‐293FT cells were transfected with OGT and the O‐GlcNAcylation of global proteins was analyzed by Western blots developed with anti‐O‐GlcNAc, RL2, and CTD110.6, and anti‐tubulin. (B) PKAcα and PKAcβ were immunoprecipitated by O‐GlcNAc antibodies. O‐GlcNAcylated proteins were immunoprecipitated by a mixture of RL2 and CTD110.6 from HEK‐293FT cells mock‐transfected (Con) or transfected with OGT (OGT) and analyzed by Western blots developed with anti‐PKAcα, anti‐PKAcβ, and RL2. (C) Immunopurified PKAcα or PKAcβ was immunoreacted by O‐GlcNAc antibodies. PKAcα or PKAcβ tagged with HA at N‐terminus was overexpressed in HEK‐293FT cells with/without co‐expression of OGT and immunoprecipitated with monoclonal anti‐HA. The immunoprecipitated complexes were analyzed by Western blots developed with a mixture of RL2 and CTD110.6 and polyclonal anti‐HA. HC, IgG heavy chain.

To learn whether PKAcs are modified by O‐GlcNAcs, we upregulated O‐GlcNAcylation by overexpression of OGT in HEK‐293FT cells (Fig. 1A) and then immunoprecipitated O‐GlcNAcylated proteins by a mixture of two O‐GlcNAc antibodies (RL2 and CTD110.6). We found that either PKAcα or PKAcβ was immunoprecipitated by O‐GlcNAc antibodies (Fig. 1B). Upregulation of O‐GlcNAcylation by overexpression of OGT increased the amount of PKAcα and cβ immunoprecipitated by O‐GlcNAc antibodies (Fig. 1B). These data suggest that PKAcα and PKAcβ are probably modified by O‐GlcNAc, and overexpression of OGT increases their O‐GlcNAcylation.

PKAcα1 and PKAcβ1 are two major isoforms of PKAc expressed ubiquitously (Skalhegg & Tasken, 2000). They have the same length and share 93% sequence identity (Soberg *et al*., 2013). To confirm the O‐GlcNAcylation of PKAcs, we overexpressed PKAcα (PKAcα1) or PKAcβ (PKAcβ1) tagged with HA at the N‐terminal in HEK‐293FT cells and immunoprecipitated them with monoclonal anti‐HA. Then, we analyzed them with Western blots developed by anti‐O‐GlcNAc. We found that immunopurified PKAcα (Fig. 1C, lane 1) or PKAcβ (Fig. 1C, lane 3) immunoreacted with O‐GlcNAc antibodies, RL2/CTD110.6 (Fig. 1C), confirming that either PKAcα or PKAcβ is an O‐GlcNAc protein. Overexpression of OGT increased the immunoreaction of PKAcα with RL2/CTD110.6 (Fig. 1C, lane 2), indicating that OGT overexpression upregulates O‐GlcNAcylation of PKAcα.

### O‐GlcNAcylation of PKAc enhances its kinase activity toward tau

**Figure 2:**
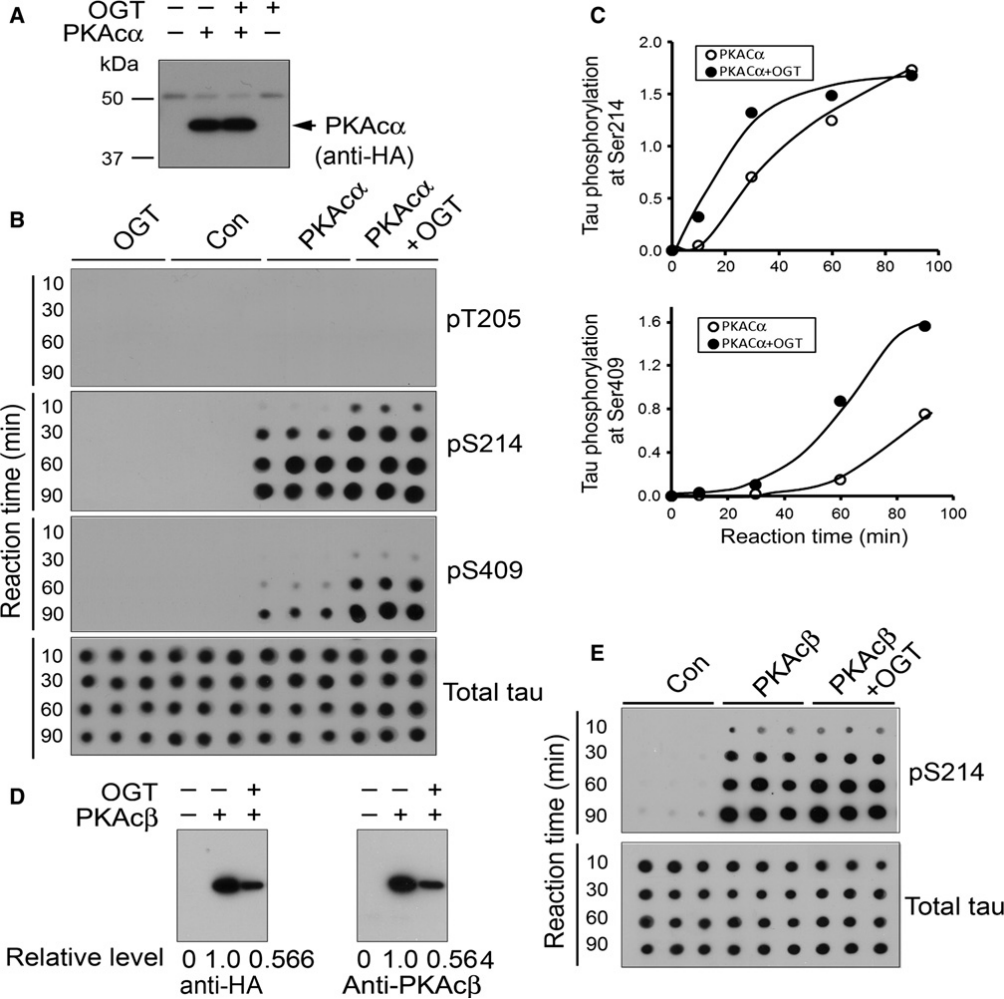
O‐GlcNAcylation enhances PKAc kinase activity toward tau. (A–C) HA‐PKAcα was expressed in HEK‐293FT cells with/without co‐expression of OGT and immunoprecipitated with monoclonal anti‐HA. Amount of the IP‐PKAcα was detected by Western blots developed with rabbit anti‐HA (A). These IP‐PKAcα were incubated with 0.2 mg mL^−1^ tau for various length of times in the reaction buffer containing 0.5 mm ATP. The phosphorylation of tau was analyzed by immunodot blots developed with anti‐pT205‐tau, anti‐pS214‐tau, anti‐pS409‐tau, and anti‐total tau (B). The level of tau phosphorylation at these sites normalized with total tau was plotted against reaction time (C). (D and E) O‐GlcNAcylation enhanced PKAcβ activity in phosphorylating tau at Ser214. HA‐PKAcβ was co‐expressed in HEK‐293FT cells with/without OGT and immunoprecipitated with anti‐HA. The immunoprecipitated‐PKAcβ was detected by Western blots developed by anti‐HA or anti‐PKAcβ and used for kinase activity assays as described in panels A and B. The phosphorylation of tau at Ser214 was assayed by immuno‐dot‐blots developed with anti‐pS214‐tau and total tau.

To study the impact of O‐GlcNAcylation on PKA kinase activity, we overexpressed PKAcα with or without OGT and immunoprecipitated PKAcα. Similar levels of PKAcα were immunoprecipitated from HEK‐293FT cells (Fig. 2A). Then, we used the immunoprecipitated PKAcα to phosphorylate tau in vitro for various lengths of time. The phosphorylation of tau was measured by immuno‐dot‐blots developed with the phosphorylation‐dependent and site‐specific tau antibodies, anti‐pT205‐tau, anti‐pS214‐tau, and anti‐pS409‐tau. We found that PKAcα phosphorylated tau at Ser214 and Ser409 effectively, but not Thr205, which is a non‐PKA target site (Fig. 2B). The PKAcα immunoprecipitated from cells that co‐expressed OGT showed greater ability in the phosphorylation of tau at both Ser214 and Ser409 (Fig. 2B,C), suggesting that upregulation of O‐GlcNAcylation by overexpression of OGT increases PKAcα activity.

To learn whether O‐GlcNAcylation also affects PKAcβ activity, we performed experiments similar to those above. The amount of immunoprecipitated‐PKAcβ from the cells that co‐expressed OGT was ~50% of the amount of PKAcβ from the cells without OGT co‐expression (Fig. 2D). However, this reduced amount of PKAcβ showed similar kinase activity as control PKAcβ to phosphorylate tau at Thr214 (Fig. 2E). Thus, similar to PKAcα, O‐GlcNAcylation was also found to enhance PKAcβ activity to tau.

### O‐GlcNAcylation regulates CREB and its phosphorylation

**Figure 3:**
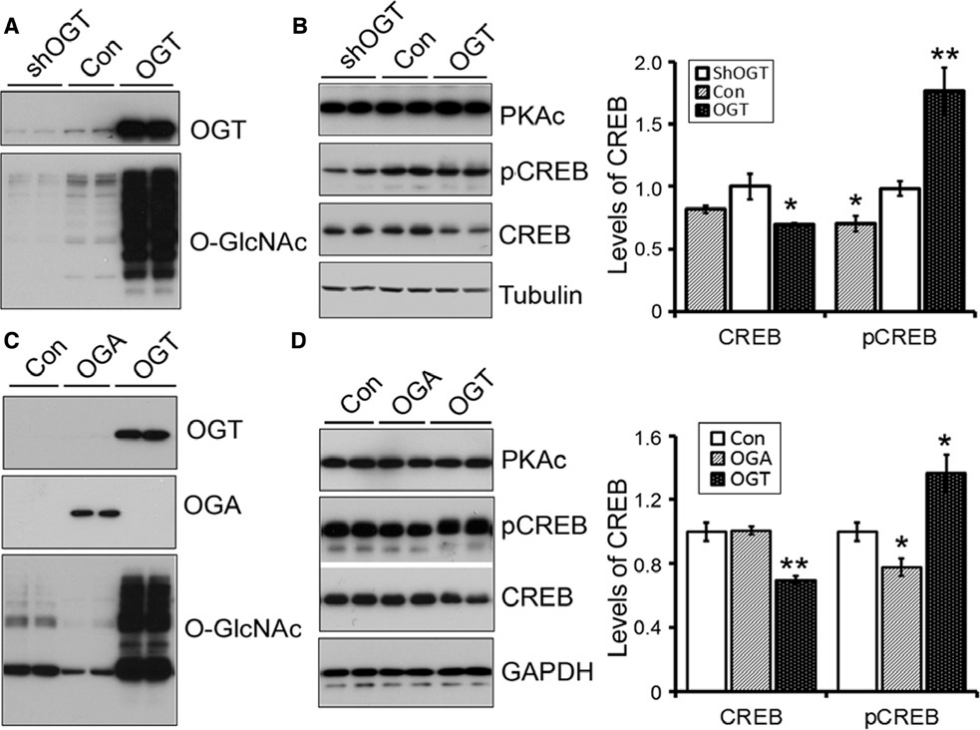
O‐GlcNAcylation affects CREB phosphorylation positively in cultured cells. HEK‐293FT cells (A, B) or N2a cells (C, D) were transfected with OGT, OGA, or shOGT. O‐GlcNAcylation and CREB phosphorylation at Ser133 were analyzed by Western blots developed with anti‐OGT, anti‐OGA, anti‐O‐GlcNAc (RL2), anti‐PKAc, anti‐pS133‐CREB, CREB, and anti‐tubulin or anti‐GAPDH 48 h after transfection. The levels of CREB and Ser133 phosphorylated CREB were normalized by the corresponding loading controls and total CREB, respectively, and presented as mean ± SD (*n* = 3); *, *P *<* *0.05; **, *P *<* *0.01.

It is well established that PKA phosphorylates CREB at Ser133 to promote its transcriptional activity (Gonzalez & Montminy, 1989). To learn the effect of PKA O‐GlcNAcylation on the phosphorylation of CREB, we altered O‐GlcNAcylation by overexpression or knockdown of OGT in HEK‐293FT cells and measured the phosphorylation of CREB at Ser133. We found that upregulation of O‐GlcNAcylation caused a reduction in CREB protein level and an increase in CREB phosphorylation at Ser133 (Fig. 3A,B). In contrast, downregulation of O‐GlcNAcylation by knockdown of OGT significantly decreased the phosphorylation of CREB, but did not affect CREB level (Fig. 3A,B). These results suggest that O‐GlcNAcylation may regulate CREB level and CREB phosphorylation at Ser133 in cultured cells. However, alteration of O‐GlcNAcylation did not affect PKAc level (Fig. 3B).

To investigate whether the regulation of CREB phosphorylation by O‐GlcNAcylation that we observed in HEK‐293FT cells above also occurs in neuron‐like cells, we overexpressed OGT or OGA to upregulate or downregulate O‐GlcNAcylation in N2a cells and then determined phosphorylation of CREB. We found that overexpression of OGT caused upward mobility of CREB in SDS‐PAGE (Fig. 3D), reduced CREB level, and increased the level of net CREB phosphorylation (Fig. 3D). Overexpression of OGA slightly decreased O‐GlcNAcylation and CREB phosphorylation (Fig. 3C,D). Like HEK‐293FT cells, N2a cells did not show any effect of O‐GlcNAcylation on the level of PKAc (Fig. 3D). These data confirm that O‐GlcNAcylation decreases CREB level and enhances CREB phosphorylation.

### O‐GlcNAcylation regulates CREB phosphorylation and alters subcellular localization of PKAcα and PKAcβ

**Figure 4:**
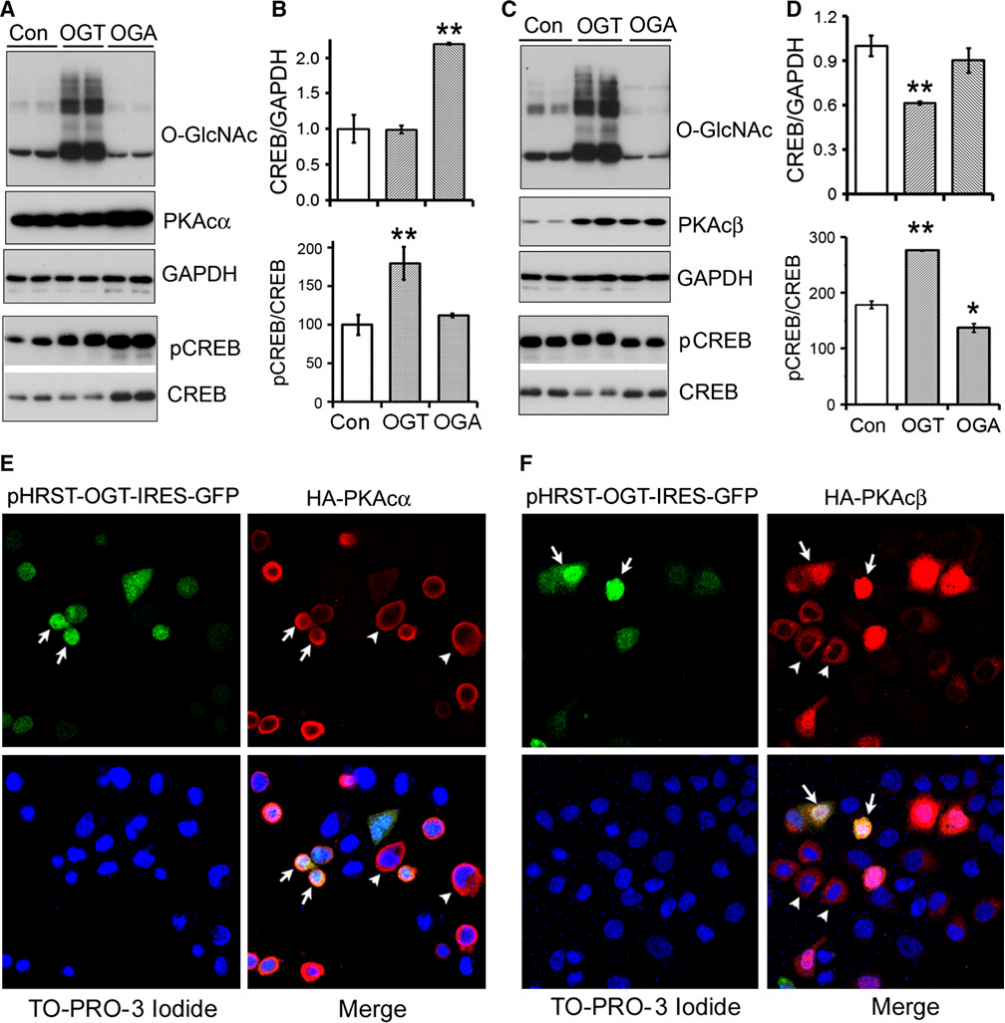
O‐GlcNAcylation promotes the subcellular translocation of PKAcs and phosphorylation of CREB. (A–D) Expression of OGT enhances the phosphorylation of CREB in PKAcα or PKAcβ co‐expressed N2a cells. N2a cells were made to co‐express HA‐PKAcα (A, B) or HA‐PKAcβ (C, D) with OGT or OGA and subjected to Western blots developed with antibodies against HA, PKAcs, CREB, pCREB, or GAPDH. Levels of CREB and phosphorylated CREB were normalized with GAPDH and CREB, respectively, and are presented as mean ± SD (*n* = 3); *P <* *0.05, **P <* *0.01. (E, F) Overexpression of OGT promoted the subcellular translocation of PKAcα (Ε) or PKAcβ (F) from cell cytoplasm to the nucleus. HA‐PKAcα or HA‐PKAcβ was co‐expressed with OGT‐GFP in HeLa cells. The cells were immunostained with anti‐HA and then stained with TO‐PRO‐3 iodide to stain nuclei. Arrow indicates a nuclear translocation of PKAcs in the cells with co‐expression of OGT‐GFP; arrow points a cytoplasmic localization of PKAcs in the cells without co‐expression of GFP‐OGT.

To determine the isoform‐specific effect of O‐GlcNAcylation of PKA on its activity in cultured cells, we co‐expressed PKAcα or PKAcβ with OGT or OGA in N2a cells and measured CREB phosphorylation. We found that alteration of O‐GlcNAcylation affected the expression of exogenous PKAcs (Fig. 4A,C). Co‐expression of OGA increased the exogenous level of either PKAcα or PKAcβ, but co‐expression of OGT increased the level of PKAcβ only, but not of PKAcα (Fig. 4A,C).

The endogenous level of CREB also was affected by co‐expression of OGT or OGA with PKAcs. Co‐expression of OGA with PKAcα (Fig. 4A,B), but not with PKAcβ (Fig. 4C,D), significantly increased CREB protein level. Co‐expression of OGT with PKAcβ (Fig. 4C,D), but not with PKAcα (Fig. 4A,B), inhibited CREB level significantly.

Regardless of CREB, co‐expression of OGT with PKAcα or PKAcβ enhanced phosphorylation of CREB. However, co‐expression of OGA with PKAcβ, but not with PKAcα, decreased the phosphorylation of CREB, even though the levels of either PKAcα or PKAcβ in these cells were increased (Fig. 4A,C). Thus, these results strongly suggest that O‐GlcNAcylation regulates phosphorylation of CREB at Ser133 positively in the cells co‐expressed with PKAcα and PKAcβ.

To study the effect of O‐GlcNAcylation on subcellular localization of PKAc, we overexpressed OGT with PKAcα or PKAcβ in HeLa cells and then investigated the subcellular localization by confocal microscopy. We observed that both PKAcα and PKAcβ were mainly located in the cytoplasm (Fig. 4E,F). In cells that co‐expressed OGT, the PKAcα was translocated to the periphery of the cell nucleus, and PKAcβ, to the nucleus (Fig. 4E,F). Thus, these data suggest that O‐GlcNAcylation regulates the subcellular localization of PKAcα and PKAcβ and promotes them to translocate to the nucleus.

### O‐GlcNAcylation regulates the phosphorylation of PKA target sites in the brain

**Figure 5:**
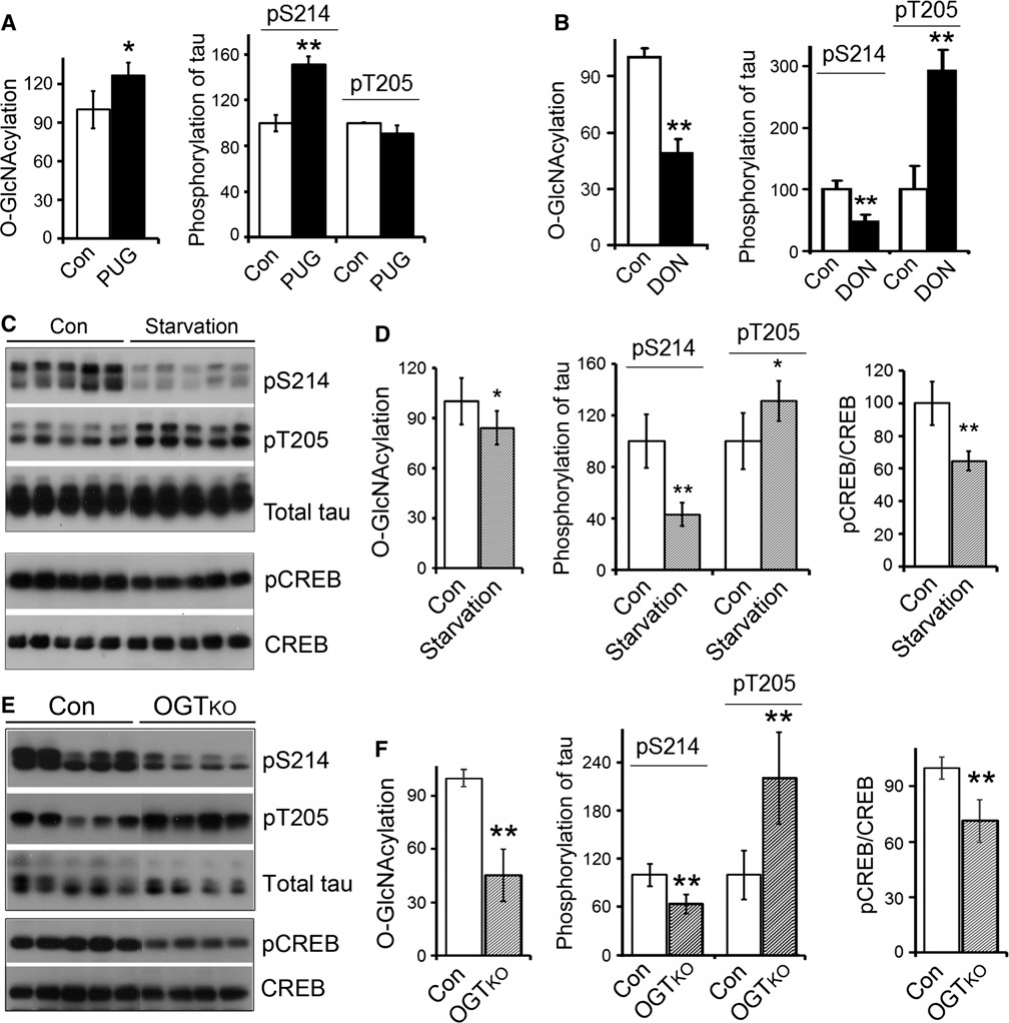
O‐GlcNAcylation affects the phosphorylation of tau at PKA target sites positively. (A) Upregulation of O‐GlcNAcylation by PUGNAC increased phosphorylation of tau at Ser214. Metabolically active rat brain slices in artificial cerebrospinal fluid were treated with PUGNAc (PUG) for 1 h and then analyzed by Western blots developed with RL2, antiphosphorylated tau at Ser214 or Thr205, and antitotal tau. (B) Downregulation of O‐GlcNAcylation suppressed tau phosphorylation at Ser214. Adult rats were intracerebroventricularly injected with 6‐diazo‐5‐oxo‐l‐norleucine (DON) and 24 h after injection that brains were analyzed by Western blots. (C, D) Starvation‐induced decrease in O‐GlcNAcylation and tau phosphorylation at Ser214. The mice were starved for 48 h, and then, brains were sacrificed for Western blots developed with antibodies against CREB, pCREB, pS214‐tau, pT205‐tau, and total tau. (E, F) Neuron‐specific knockout of OGT decreased global O‐GlcNAcyaltion and phosphorylation of CREB and tau at PKA‐target sites. The O‐GlcNAcylation, CREB phosphorylation, and tau phosphorylation in the hippocampi from the mice in which OGT was specifically knockout (OGT_KO_) were analyzed by Western blots. The levels of O‐GlcNAcylation of global proteins and phosphorylated proteins were normalized with GAPDH or total corresponding protein. Data are presented as mean ± SD (*n* = 3–4 for PUG or DON treatment, *n* = 6–7 for starvation); *, *P *<* *0.05; **, *P *<* *0.01.

To study whether upregulation of O‐GlcNAcylation affects phosphorylation of tau at the PKA target site, Ser214, in brain tissue, we treated metabolically active brain slices with the OGA inhibitor *O*‐(2‐acetamido‐2‐deoxy‐d‐glucopyranosylidenamino) *N*‐phenylcarbamate (PUGNAc) to upregulate O‐GlcNAcylation and then assayed tau phosphorylation at Ser214 (PKA site) and Thr205 (non‐PKA site as a control) by Western blots. We found that upregulation of O‐GlcNAcylation by PUGNAc (Fig. 5A) increased tau phosphorylation at Ser214, but not at Thr205 (Fig. 5A), confirming that O‐GlcNAcylation can increase PKA activity in the brain tissue, resulting in increase of tau Ser214 phosphorylation.

UDP‐GlcNAc is the donor substrate of O‐GlcNAcylation, which is synthesized through the hexosamine biosynthetic pathway (HBP) from glucose. Glutamine:fructose‐6‐phosphate amidotransferase (GFAT) is the rate‐limiting enzyme of this pathway. We injected a specific inhibitor of GFAT, 6‐diazo‐5‐oxonorleucine (DON), intracerebroventricularly to downregulate O‐GlcNAcyaltion of proteins in rat brains (Liu *et al*., 2009). We found that DON significantly suppressed protein O‐GlcNAcylation in rat brains (Fig. 5B). The phosphorylation of tau at the PKA site―Ser214―was decreased, but at a non‐PKA site―Thr205― was increased (Fig. 5B), the latter of which is due to a different mechanism (Liu *et al*., 2009). These results are consistent with the positive regulation of PKA activity by O‐GlcNAcylation.

Low glucose uptake causes downregulation of the O‐GlcNAcylation of brain proteins (Liu *et al*., 2004, 2009). To study whether starvation‐induced lower brain glucose uptake suppresses PKA activity, resulting in decrease in the phosphorylation of PKA target substrates, we starved mice for 48 h and then assayed the phosphorylation of tau at Ser214 and CREB at Ser133. We found that starvation decreased global O‐GlcNAcylation (Fig. 5D). The phosphorylation of Ser214 of tau and Ser133 of CREB was reduced significantly, but tau Thr205 phosphorylation was dramatically increased in the mice after 48 h starvation (Fig. 5C,D). These data suggest that phosphorylation of PKA target sites is associated with O‐GlcNAcylation positively.

To further confirm the regulation of PKA by O‐GlcNAcylation, we downregulated O‐GlcNAcylation by neuron‐specific knockout of OGT in mice (OGT_KO_) and then determined the phosphorylation of tau and CREB. We found that OGT knockout caused a dramatic reduction in O‐GlcNAcylation (Fig. 5F). The phosphorylation of PKA target sites, Ser133 of CREB and Ser214 of tau, was significantly decreased in OGT_KO_ mouse brains (Fig. 5E,F), confirming that reduced O‐GlcNAcylation may downregulate PKA signaling, leading to a decrease in phosphorylation of PKA target sites.

### Downregulation of O‐GlcNAc by DON impairs learning and memory

**Figure 6:**
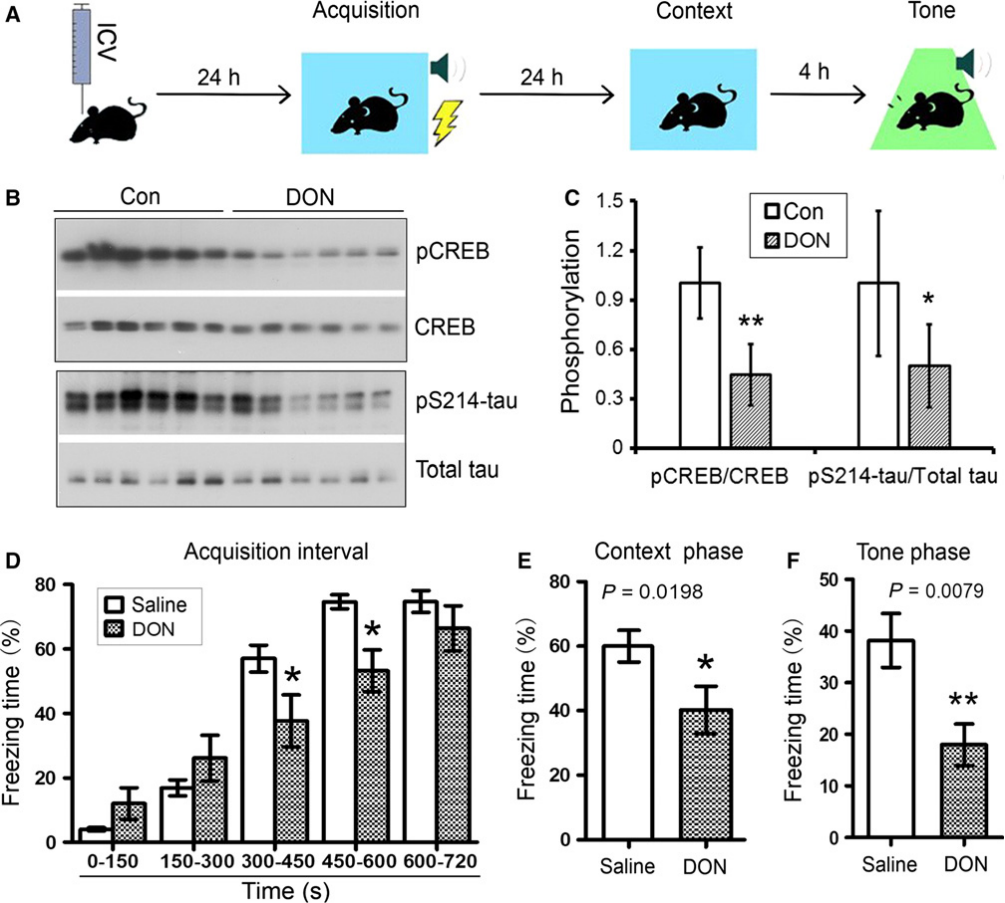
DON downregulates PKA‐CREB signaling and impairs learning and memory in mice. Schematic of fear conditioning test. The mice were injected intracerebroventricularlly with DON and trained with tone–electric shock pairings for four trials 24 h after injection. One day after training phase, the mice were subjected to context text in the same context chamber and cued tone text in different chamber but with tone. (B, C) Phosphorylation of CREB and tau at PKA‐target sites was decreased in DON‐treated mice hippocampi. Phosphorylation of CREB and tau in the hippocampi from DON‐treated mice 24 h after injection was analyzed by Western blots (B). The level of CREB phosphorylated at Ser133 or tau at Ser214 was normalized with the corresponding protein (C). Data are presented as mean ± SD (*n* = 6). *, *P *<* *0.05; **, *P *<* *0.01. (D, F) Learning and memory were impaired in mice injected with DON. The mice were subjected to the fear conditioning test 24 h after intracerebroventricular injection with DON. The freezing time was recorded during training phase (D) and test phase (E, F). Percentage of freezing are presented as mean ± SD (*n* = 12–13); *P *<* *0.05; **P <* *0.01.

PKA‐CREB signaling plays an important role in learning and memory. Extensive studies have shown that CREB is not only a requirement but also a critical driving force for the consolidation of long‐term conditioned fear memories (Bourtchuladze *et al*., 1994; Kida *et al*., 2002; Viosca *et al*., 2009; Zhou *et al*., 2009). We investigated whether decreased PKA‐CREB signaling induced by decreased O‐GlcNAcylation affects auditory fear conditioning in mice. Because knockout of OGT causes significant neuron loss (unpublished observation), we here downregulated O‐GlcNAcylation by intracerebroventricular injection of DON, after which the mice were subjected to the fear conditioning test 24 h after injection (Fig. 6A). We found that, in addition to reduction in tau phosphorylation at Ser214 as seen in Fig. 5B, the level of CREB phosphorylated at Ser133 was significantly decreased in DON‐treated mouse brains (Fig. 6B,C), suggesting that PKA activity is downregulated. During the training phase, DON‐treated mice did not show abnormal responses to the shock. The freezing time was not significantly different between DON‐treated mice and saline‐treated mice at the first and the second shocks; the freezing time was dramatically reduced in DON group as compared with saline group at the third and fourth shocks; and these two groups of mice showed similar freezing time at the last shock (Fig. 6D), suggesting learning impairment in DON‐treated mice. In the context and cued tone phase, DON‐treated mice showed less freezing time than saline‐treated mice, suggesting that memory in these mice also was impaired (Fig. 6E,F). Thus, this fear conditioning test indicated that learning and memory were impaired in DON‐treated mice.

## Discussion

Protein kinase A is one of the most important protein kinases involved in the regulation of multiple cellular functions. The best‐studied regulatory mechanism of PKA is its interaction with cAMP, which leads to the dissociation of regulatory subunits from the catalytic subunits, resulting in its activation. Phosphorylation of PKAc at Thr197 is required for PKA's maximal activity. In the present study, we found, for first time, that PKAcα and PKAcβ are both modified by O‐GlcNAc, which enhances its kinase activity toward tau. Alteration of O‐GlcNAcylation by both molecular and pharmacological approaches was found to positively regulate the phosphorylation of PKA target sites, tau at Ser214 and CREB at Ser133, in cultured cells and *in vivo*, suggesting that PKA activity is modulated by its O‐GlcNAcylation. In AD brain, decreased O‐GlcNAcylation is accompanied by unchanged phosphorylation of tau at Ser214 and increased phosphorylation of tau at Thr205. Downregulation of O‐GlcNAcylation in mouse brains by intracerebroventricular injection of DON causes reduction in phosphorylation of CREB and of tau at PKA target sites and learning and memory deficits. Thus, O‐GlcNAcylation may be a new way to regulate PKA activity. PKA signaling is involved in learning and memory via phosphorylation of CREB at Ser133.

O‐GlcNAcylation is regulated by OGT and OGA, which are enriched in brain (Kreppel *et al*., 1997; Gao *et al*., 2001; Okuyama & Marshall, 2003; Love & Hanover, 2005). Mouse brain‐specific deletion of OGT results in perinatal lethality associated with severe motor defects (O'Donnell *et al*., 2004). KCl‐induced depolarization in cultured cells rapidly activates OGT and increases O‐GlcNAc levels, suggesting the involvement of dynamic O‐GlcNAc modifications in neuronal signaling (Song *et al*., 2008). Pharmacological elevation of O‐GlcNAc levels *in vivo* enhances long‐term potentiation (LTP), whereas reduction in O‐GlcNAc levels blocks LTP (Tallent *et al*., 2009). While upregulation of O‐GlcNAcylation pharmacologically prevents cognitive decline and memory impairment in AD transgenic mice (Kim *et al*., 2013; Yuzwa *et al*., 2014). In the present study, we found that PKAc is modified by O‐GlcNAc. PKAcα or PKAcβ immunopurified from OGT‐overexpressed cells had higher kinase activity in the phosphorylation of tau. Upregulation or downregulation of O‐GlcNAcylation by molecular biological or pharmacological approaches increased or decreased the phosphorylation of PKA target sites in cultured cells and/or *in vivo*, suggesting the corresponding alterations of PKA activity to the changes in O‐GlcNAcylation. Activated PKA phosphorylates CREB, a key regulator in learning and memory.

In addition to phosphorylate tau and CREB, it is reported that PKA also phosphorylates glycogen synthase kinase‐3β (GSK‐3β) at Ser9 (the inactive form of the enzyme) and inhibits its activity (Fang *et al*., 2000). GSK‐3β phosphorylates CREB at Ser129, which Ser133 phosphorylation is required for its phosphorylation by GSK‐3β (Fiol *et al*., 1994) and suppressed its DNA binding activity (Grimes & Jope, 2001). Moreover, O‐GlcNAcylation also modulates PI3K‐AKT‐GSK‐3β pathway (Shi *et al*., 2012). Thus, O‐GlcNAcylation may modulate CREB signaling through PKA and GSK‐3β pathways.

CREB regulates many neuronal processes, including brain development, circadian rhythm, and long‐term memory. Phosphorylation of CREB at Ser133 leads to recruitment of the coactivator CREB‐binding protein and activation of CREB‐mediated transcription (Chrivia *et al*., 1993). It has been reported that CREB is also O‐GlcNAcylated at Ser40 and Thr227 or Thr228 (Rexach *et al*., 2012); and Thr259, Ser260, or Thr261 (Lamarre‐Vincent & Hsieh‐Wilson, 2003), but Ser40 is the predominant O‐GlcNAcylation site (Rexach *et al*., 2012). CREB can be modified simultaneously by O‐GlcNAc at Ser40 and by O‐phosphate at Ser133. However, glycosylation at Ser40 does not affect the amount of Ser133 phosphorylation that occurs in response to neuronal depolarization, but glycosylation functions to repress CREB activity (Rexach *et al*., 2012). Overexpression of S40A CREB (prevents O‐glycosylation) in mouse lateral amygdala significantly enhances memory by 2 h after training compared to mice that overexpress wild‐type CREB, and the enhanced memory persisted at 24 h. The expression of S40A CREB in the amygdala may promote more rapid long‐term memory consolidation than the expression of wild‐type CREB. O‐GlcNAcylation at Ser40 inhibits CREB activity in the formation of long‐term memory (Rexach *et al*., 2012). Interestingly, Ser133 phosphorylation is required for the induction of Ser40 O‐GlcNAcylation by depolarization, but forskolin‐mediated stimulation of Ser133 phosphorylation via PKA has no effect on the CREB O‐GlcNAcylation. In the present study, we found that downregulation of O‐GlcNAcylation by DON impairs learning and memory accompanied by downregulation of PKA‐CREB signaling.

Alzheimer's disease is clinically characterized by learning and memory deficits. In AD brain, O‐GlcNAcylation is downregulated as a result of impaired glucose uptake (Liu *et al*., 2004, 2009). The phosphorylation of tau at other sites all increased significantly, but not of tau Ser214, suggesting downregulation of PKA activity. Several studies suggest impaired signaling of PKA‐CREB in AD brain (Yamamoto‐Sasaki *et al*., 1999; Puzzo *et al*., 2005; Liu *et al*., 2006). CREB is rapidly dephosphorylated during the postmortem interval (Wang *et al*., 2015), but we could not obtain reliable results on the level of CREB phosphorylation in AD brain. Moreover, we recently found that PKA‐CREB signaling regulates the expression of neuron‐specific glucose transporter III (GLUTIII) (Jin *et al*., 2013). Thus, downregulation of PKA‐CREB signaling results from glucose uptake impairment via decreased O‐GlcNAcylation that further suppresses GLUTIII expression, which further suppresses O‐GlcNAcylation and PKA activity by a positive feedback mechanism, and finally, causes learning and memory deficits in AD.

CREB is degraded by the ubiquitin–proteasome system pathway (Costes *et al*., 2009). Phosphorylation has been found to control proteasome degradation. Phosphorylation induced by hypoxia at non‐PKA site targets CREB to the ubiquitin–proteasome pathway (Costes *et al*., 2009). Forskolin significantly increased phosphorylation of CREB at Ser133 (Shi *et al*., 2011; Jin *et al*., 2013), but does not affect its protein level, suggesting that phosphorylation of CREB at Ser133 is not involved in CREB degradation. CREB is also modified by O‐GlcNAc. In the present study, we found that OGT or OGA overexpression significantly decreased or increased the level of CREB, respectively, suggesting that O‐GlcNAcylation of CREB may regulate its degradation, which remains elusive.

In summary, PKAcs are modified by O‐GlcNAc, resulting in an increase in its kinase activity, which is a novel way to regulate PKA activity. Downregulation of O‐GlcNAcylation causes a decrease in PKA‐CREB signaling and learning and memory deficits. Lower glucose uptake in AD brain may cause suppression of O‐GlcNAcylation‐modulated PKA‐CREB signaling and thus could be involved in learning and memory deficits in this disease. Increased O‐GlcNAcylation might be a therapeutic approach to prevent the learning and cognitive impairment in AD.

## Experimental procedures

### Animals

Sprague Dawley (SD) rats and C57BL/6NJCL and FVB mice were purchased from Charles River Laboratories (Kingston, NY, USA) and Nanjing Animal Model Center (Nanjing, JS, China). The animals were housed on a 12‐h light/dark schedule with free access to food and water. Animal use was in full compliance with the National Institutes of Health guidelines and was approved by our Institutional Animal Care and Use Committee.

The Cre^(+)^;ogt^loxp(+)/loxp(+)^ mice were generated by crossing hemizygous B6.129S6‐Tg (Camk2α‐cre/ERT2)1Aibs/J Cre‐expressing mice with B6.129‐Ogttm1Gwh/J OGT‐floxed mice, both of which were purchased from the Jackson Laboratory (Bar Harbor, ME, USA), and backcrossed to C57BL/6J mice for at least nine generations in our animal colony. The neuronal OGT knockout (OGT_KO_) in these mice was induced by intraperitoneal (i.p.) injection of tamoxifen (75 mg kg^−1^ day^−1^ for four consecutive days) or, as a control, vehicle (corn oil containing 10% ethanol). The mice were killed by cervical dislocation, and the brains were removed 9 days after tamoxifen injection. The cerebral cortices were immediately dissected and homogenized with prechilled homogenizing buffer (50 mm Tris–HCl, pH 7.4, 50 mm GlcNAc, 20 μm UDP, 2.0 mm EGTA, 2. 0 mm EDTA, 2 mm Na_3_VO_4_, 50 mm NaF, 1 mm AEBSF, 10 μg mL^−1^ aprotinin, 10 μg mL^−1^ leupeptin, and 10 μg mL^−1^ pepstatin A).

### Plasmids, proteins, and antibodies

pRK172 containing the largest isoform of human tau, pRK172/tau_441_, was kindly provided by Dr. Michel Goedert (Molecular Biology Unit, Medical Research Council, Cambridge, UK). pHRST‐OGT‐IRES‐GFP, pCI/OGT, pCI/PKAcα, and pCI/PKAcβ were constructed as described previously, and their sequences were confirmed (Shi *et al*., 2011, 2012). Recombinant tau_441_ was expressed and purified from *Escherichia* *coli* in our laboratory. The primary antibodies used in this study are listed in Table 1. Horseradish peroxidase (HRP)‐conjugated anti‐mouse and anti‐rabbit IgG were obtained from Jackson ImmunoResearch Laboratories (West Grove, PA, USA). Enhanced chemiluminescence (ECL) kit was from Thermo Scientific (Rockford, IL, USA).

**Table 1:** Primary antibodies employed in this study

Antibody	Type	Specificity	Phosphorylation site/epitope	Reference/source
R134d	Poly‐	Tau	N/A	Pei *et al*. (1998)
43D	Mono‐	Tau	6–18	Biolegend, San Diego, CA, USA
Anti‐pT205‐tau	Poly‐	p‐tau	pThr205	Invitrogen
Anti‐pS214‐tau	Poly‐	p‐tau	pSer214	Invitrogen
Anti‐pS409‐tau	Poly‐	p‐tau	pThr409	Invitrogen
RL2	Mono‐	O‐GlcNAcylated proteins	O‐GlcNAc	Affinity BioReagent, Golden, CO, USA
CTD110.6	Mono‐	O‐GlcNAcylated proteins	O‐GlcNAc	Santa‐Cruz, Santa Cruz, CA, USA
Anti‐pS133‐CREB	Poly‐	p‐CREB	pSer133	Cell Signaling, Danvers, MA, USA
Anti‐CREB	Poly‐	CREB		Invitrogen
Anti‐PKAcα	Poly‐	PKAcα		Santa‐Cruz
Anti‐PKAcβ	Poly‐	PKAcβ		Santa‐Cruz
Anti‐OGT	Poly‐	OGT		Sigma
Ant‐HA	Poly‐	HA		Sigma
Anti‐HA	Mono‐	HA		Sigma
Anti‐tubulin	Mono‐	Tubulin		Sigma
Anti‐GAPDH	Poly‐	GAPDH		Santa‐Cruz

GAPDH, glyceraldehyde‐3‐phosphate dehydrogenase; mono‐, monoclonal; p‐, phosphorylated; Poly‐, polyclonal; Ser, serine; Thr, threonine.

### Cell culture and transfection

Human embryonic kidney cell line (HEK‐293FT), mouse neuroblastoma cell line (N2a), and human cervix epithelia cell line (HeLa) were cultured in Dulbecco's modified Eagle's medium supplemented with 10% fetal bovine serum, 100 U mL^−1^ penicillin, and 100 U mL^−1^ streptomycin and incubated in a humidified atmosphere containing 5% CO_2_ at 37 °C.

Transfection of the cultured cells was performed using FuGENE 6 and FuGENE HD (Promega, Madison, WI, USA) according to the manufacturer's instructions. Lipofectamine^™^2000 reagent (Invitrogen, Carlsbad, CA, USA) was used for the transfection of HeLa cells according to the manufacturer's instructions. For knockdown of OGT expression, we used SureSilencing shRNA plasmids for human OGT under the control of the U1 promoter (SA Bioscience, Frederick, MD, USA). After transfection for 2 days, cells were lysed, and the cell lysates were analyzed using Western blots.

### Immunoprecipitation

Cultured cells were washed twice with PBS and then lysed in the lysate buffer (50 mm Tris–HCl, pH 7.4, 150 mm NaCl, 50 mm NaF, 1 mm Na_3_VO_4_, 0.1% Triton X‐100, 1% NP40, 0.25% sodium deoxycholate, 5 mm AEBSF, 10 μg mL^−1^ leupeptin, 10 μg mL^−1^ aprotinin, and 10 μg mL^−1^ pepstatin). Insoluble materials from cultured cell lysates were removed by brief centrifugation at 4 °C, and the supernatants were incubated with the immunoprecipitating antibody precoupled onto protein G beads at 4 °C overnight. The beads were washed with the lysate buffer twice and then with TBS twice. The bound proteins were eluted by boiling the beads in Laemmli sample buffer. The samples were analyzed by Western blots.

### Western blots

For Western blots, cultured cells were lysed in Laemmli SDS sample buffer directly, and brain homogenates in buffer (50 mm Tris–HCl, pH 7.4, 8.5% sucrose, 50 mm NaF, 1.0 mm Na_3_VO_4_, 10 mm β‐mercaptoethanol, 2.0 mm EDTA, 5 mm AEBSF, 10 μg mL^−1^ leupeptin, 10 μg mL^−1^ aprotinin, and 10 μg mL^−1^ pepstatin) were diluted in 2× Laemmli SDS sample buffer at 1:1 ratio, followed by heating at 95 °C for 5 min. Proteins were first resolved in SDS‐PAGE, and the proteins in the gels were transferred onto Immobilon membrane (Millipore, Billerica, MA, USA), followed by incubation with primary antibody, washing and incubation with HRP‐conjugated secondary antibodies. The protein–antibody complexes were visualized by the Pierce ECL Western Blotting Substrate (Thermo Scientific) and exposed to Kodak medical X‐ray film (Denville Scientific Inc, Holliston, MA, USA). Unrelated lanes were removed and specific immunostaining was quantified using the multi gauge software V3.0 from Fuji Film (Santa Clara, CA, USA).

### Immunoaffinity purification of HA‐tagged PKAc at N‐terminus (HA‐PKAc) from HEK‐293FT cells

HEK‐293FT cells were co‐transfected with pCI/HA‐PKAcα or pCI/PKAcβ and pCI/OGT and lysed with lysis buffer 48 h after transfection. Insoluble materials were removed by brief centrifugation at 16 000 *g*. The supernatant was incubated with anti‐HA antibody preconjugated onto protein G beads overnight at 4 °C. The beads were washed twice with lysis buffer and twice with TBS, and affinity‐purified HA‐PKAcα or PKAcβ was detected by Western blots.

### PKA kinase activity assay

To measure the activity of PKA, recombinant tau_441_ (0.2 mg mL^−1^) was incubated with the immunopurified PKA above in the buffer consisting of 40 mm HEPES (pH 6.8), 10 mm β‐mercaptoethanol, 10 mm MgCl_2_, 1.0 mm EGTA, and 0.2 mm ATP at 30 °C for various periods. The reaction was stopped by adding acetic acid. The reaction products were subjected to immuno‐dot‐blot analysis for the site‐specific phosphorylation of tau, as described previously (Liu *et al*., 2002).

### Immuno‐dot‐blot analyses

To measure the level of phosphorylated tau, the samples were diluted serially with 0.2% BSA in TBS containing 50 mm NaF, 1 mm Na_3_VO_4_, and 2 μg mL^−1^ each of aprotinin, leupeptin, and pepstatin and applied onto nitrocellulose membrane (Schleicher and Schuell, Keene, NH, USA) at 5 μL per grid (7 × 7 mm). The blot was placed in a 37 °C oven for 1 h to allow the protein to bind to the membrane and was processed as described above for Western blots.

### Immunocytochemistry

HeLa cells were plated on coverslips in 24‐well plates 1 day before transfection at 50–60% confluence and then transfected with pHRST‐OGT‐IRES‐GFP, as described above. After washing with PBS, the cells were fixed with 4% paraformaldehyde in PBS for 30 min at room temperature, blocked with 10% goat serum in 0.2% Triton X‐100, PBS for 2 h at 37 °C, and incubated with mouse anti‐PKAc (1:50) overnight at 4 °C. The cells were then washed and incubated for 1 h with secondary antibody (Alexa 488‐conjugated goat anti‐mouse IgG, 1:1000) plus TO‐PRO‐3 iodide at room temperature. The cells were washed with PBS, mounted with Fluoromount‐G, and visualized with a confocal microscope (Nikon, Melville, NY, USA).

### Starvation of mice

Three‐month‐old male C57BL/6NJCL mice (Charles River Laboratories) were housed singly in cages with grid floor to prevent coprophagy. After the mice had no access to food but had access to water for 48 h, they were sacrificed by cervical dislocation, and the cerebral cortices were immediately dissected and stored at −80 °C until use.

### Metabolically active rat brain slices

Metabolically active brain slices (400 μm × 400 μm) were prepared from 6.5‐month‐old male Wistar rats and incubated in oxygenated artificial cerebrospinal fluid (CSF), as described (Gong *et al*., 2000). 0.2 mm *O*‐(2‐acetamido‐2‐deoxy‐d‐glucopyranosylidene) amino‐*N*‐phenylcarbonate (PUGNAc) was added to the artificial CSF during incubation. After 1‐h incubation, the slices were homogenized and analyzed by Western blots.

### Intracerebroventricular injection

Sprague Dawley rats or FVB mice were first anesthetized by intraperitoneal injection of chloral hydrate (30 mg kg^−1^ for rats) or 2.5% tribromoethanol (Avertin) (Sigma‐Aldrich, St. Louis, MO, USA) (20 mL kg^−1^ for mice) and then placed on a stereotactic instrument with the incisor bar. After the scalp was incised and retracted, a 10‐μL syringe (Hamilton, Reno, NV, USA) was used to stereotactically inject compounds into the lateral ventricle of the cerebrum according to stereotaxic coordinates (bregma and dura of anterior–posterior −0.8 mm, left lateral 1.2 mm, and dorsal–ventral −3.5 mm for rats, and bregma and dura of anterior/posterior −0.5 mm, left lateral 1.0 mm, and dorsal/ventral −2.5 mm for mice). A total of 10 μL of 5 mm for rats and 2 μL of 50 μm for mice of DON dissolved in artificial cerebrospinal fluid (aCSF) (140 mm NaCl, 3.0 mm KCl, 2.5 mm CaCl2, 1.0 mm MgCl2 and 1.2 mm Na2HPO4, pH 7.4) was injected into the left ventricle of the brain. The same volume of aCSF was injected into the left ventricle for control animals. Sacrifice by cervical dislocation occurred 24 h after injection for rats or 60 h after injection for mice. The brains were immediately removed and stored at −80 °C. One cohort of mice was subjected to fear conditioning test 24 h after injection.

### Contextual and cued fear conditioning

Before experiment, mice were habituated in the testing room for 3 days, 3 h each time. In the training session, on day 1, mice were placed in the testing chamber for a total of 720 s. Baseline was recorded from 0 to 120 s, and then, four tone–shock pairings were applied. A tone (30 s, 75 dB, 2000 Hz) was turned on at 120 s, 270 s, 420 s, and 570 s, each of which co‐terminated with a foot shock (2 s, 0.6 mA). In the contextual fear session, on day 2, mice were placed into the same testing chamber and scored for percentage of freezing for 5 min, during which time no tone or shock was given. In cued conditioning session 4 h after contextual testing, the mice were introduced to the same testing chamber with altered context. After a 180‐s baseline habitation, tone was given for 180 s without the shock pairing. The percent of time the mouse spent freezing was recorded during the tone phase and was used as a measure of cued fear conditioning. All the data were collected and analyzed with the Freeze Frame and Freeze View system (Coulbourn Instruments, Whitehall, PA, USA).

### Statistical analysis

Data are presented as mean ± SD and analyzed by the unpaired two‐tailed Student's *t*‐test or Mann–Whitney *U*‐test (for the data with non‐normal distribution) for two‐group comparison and by one‐way ANOVA for multiple‐group analysis.

## Author contributions

S.X, N. J., J.G., J.S., D.C., J.S., L.Z, C.D., and J.G. performed experiments and analyzed data; C.X.G. and K.I. provided reagents and reviewed the manuscript critically; and F.L. performed kinase activity assay and immunoblot analysis, designed the study, analyzed data, and wrote the manuscript.

## Conflict of interest

None declared.

## Funding

No funding information provided.
